# Head and Neck Muscle Activity in Post‐Stroke Dysphagia Patients: Muscle Dynamics and Implications for Rehabilitation

**DOI:** 10.1111/joor.70044

**Published:** 2025-08-20

**Authors:** Yingying Zhang, Mingyuan Wu, Weixia Yu, Yinuo Dai, Zhina Gong, Chunyan Niu, Jianzheng Cai

**Affiliations:** ^1^ Department of Radiotherapy The First Affiliated Hospital of Soochow University Suzhou China; ^2^ Medical College of Yangzhou University Yangzhou China; ^3^ Department of Nursing The First Affiliated Hospital of Soochow University Suzhou China

**Keywords:** muscular function, phonatory tasks, post‐stroke dysphagia, rehabilitation, surface electromyography

## Abstract

**Background:**

This study utilised surface electromyography (sEMG) to evaluate the activity patterns of head and neck muscle groups in patients with post‐stroke dysphagia (PSD), aiming to establish a scientific basis for muscle function rehabilitation in these individuals.

**Methods:**

A case–control study, which involved 60 PSD patients and 60 healthy volunteers, was conducted between 1 August 2022, and 28 February 2024. The sEMG signals of head and neck muscles were recorded during dry swallowing and various phonatory tasks, including pursed‐lip breathing (PLB), end‐inspiratory breath holding (EIBH), lip trills and the pronunciation of the sounds /a/, /n/ and /m/. The mean sEMG amplitude of each muscle group during these tasks was measured and analysed.

**Results:**

For the PSD patients, the sEMG signal of the masseter muscle (MS) during dry swallowing was significantly correlated with its signal recorded during all tasks except PLB. Similarly, the sEMG signal of the orbicularis oris muscle (OO) during dry swallowing showed significant correlation with its signal during PLB, /a/ and /n/ tasks, whereas for the submental muscle (SUB), significant correlation existed across all tasks. In the healthy controls, the sEMG signal of MS and OO during dry swallowing did not correlate significantly with their signals recorded during any task, whereas for SUB, significant correlation existed between the sEMG signal during dry swallowing and the signals recorded during lip trill, /a/, /n/ and /m/ tasks. Across all subjects, the sEMG signal of the sternocleidomastoid muscle (SCM) during dry swallowing was significantly correlated with its signal recorded during all tasks. According to multiple regression analyses, the following tasks affected certain muscle groups in the patients but not in the controls: lip trills on MS, SUB and SCM, pronouncing /a/ on OO and pronouncing /n/ on SCM.

**Conclusion:**

For the PSD patients, there were varying levels of muscle activation in head and neck muscles during different phonatory tasks. Carefully selecting phonatory tasks may help develop targeted rehabilitation strategies.

## Graphical abstract

Surface electromyography (sEMG) was used to collect the electromyographic signals of the head and neck muscles during swallowing and phonatory tasks, revealing the differences in muscle activation levels of patients with dysphagia under different phonatory tasks.

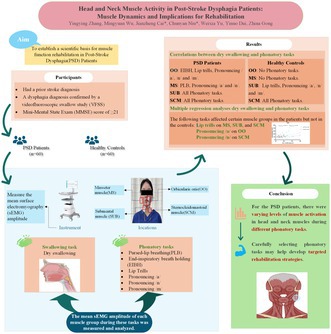

**Abbreviations**
AMR: alternating movement rate
DOSS: Dysphagia Outcome and Severity Scale
EIBH: end‐inspiratory breath holding
LSVT: Lee Silverman Voice Treatment
MMSE: mini‐mental state exam score
MPT: maximal phonation time
MS: masseter muscle
OO: orbicularis oris muscle
PLB: pursed‐lip breathing
PSD: post‐stroke dysphagia
SCM: sternocleidomastoid muscle
sEMG: surface electromyography
SUB: submental muscle
VFSS: videofluoroscopic swallow study

## 1 Introduction

Stroke remains one of the leading causes of death and disability worldwide, posing a significant threat to public health [1]. Post‐stroke dysphagia (PSD), a common condition following stroke, affects a substantial proportion of patients, and its prevalence is estimated to be 38.57% by Wen et al. [2] and 46.6% by Song et al. [3]. It is characterised by impaired swallowing safety and efficiency due to deficits in coordination, strength and sensation in the head and neck muscles involved in swallowing. These impairments result from neurological damage affecting the nerves that regulate swallowing functions [4]. Post‐stroke dysphagia is linked to serious complications, such as aspiration pneumonia, which significantly increases morbidity and mortality [5, 6, 7, 8]. Beyond its health impact, dysphagia and its related complications significantly reduce patients' quality of life and impose considerable economic burdens on healthcare systems [9, 10].

Swallowing is a complex process involving multiple muscle groups in the head and neck. The orbicularis oris muscle (OO) facilitates lip closure, while the masseter muscle (MS) contracts alternately to enable chewing. During the pharyngeal phase of swallowing, a series of coordinated actions protect the airway, including vocal fold contraction and the elevation of the hyoid bone and larynx by the submental muscle group (SUB). The sternocleidomastoid muscle (SCM) contributes to head and neck posture, providing stability that supports the complex coordination of muscles involved in swallowing. This stabilisation helps maintain postural control during swallowing, particularly in individuals with dysphagia who may need additional neck muscle engagement [11]. Damage to these muscle groups can result in various swallowing difficulties, such as food leakage from the lips, inadequate mastication and accidental aspiration of food into the airway [12, 13].

Vocalisation and swallowing both rely on precise neuromuscular control in the head and neck, and neurological damage can impair both systems [14]. The peripheral vocal control system integrates motor functions of the laryngeal and oral‐facial musculature, receiving input from bilaterally coordinated motor areas in the brain [15]. Producing normal vocalisation requires synchronised actions of the lips, jaw, tongue and larynx, along with proper airflow regulation. The laryngeal motor cortex, in conjunction with brainstem central pattern generators, plays a crucial role in coordinating these movements for both speech and swallowing. Additionally, the pharynx, oral cavity and nasal cavity serve as resonating chambers, shaping vocal tone and intensity by filtering sound waves. Damage to these structures can lead to both dysphagia and voice disorders, highlighting the interconnected nature of vocalisation and swallowing functions [16].

Systematic anatomical research and the principle of neuroplasticity support the idea that vocalisation and swallowing functions interact dynamically, as well as the notion that therapeutic improvements in one may positively influence the other [17, 18]. Speech and swallowing share overlapping neural pathways, particularly in the sensorimotor cortex and brainstem central pattern generators, allowing targeted interventions to enhance both functions. Various vocal exercises have been shown to significantly enhance swallowing physiology and function in patients with dysphagia. For example, the Lee Silverman Voice Treatment (LSVT) improves vocal intensity and swallowing safety, particularly in individuals with Parkinson's disease [19], while therapeutic singing strengthens laryngeal control and respiratory coordination [20]. Clinical studies have employed intensive vocalisation exercises that emphasise movement intensity and sensory recalibration. These exercises target both the intrinsic and extrinsic laryngeal muscles, aiming to improve coordination between myoelectric and aerodynamic laryngeal forces. The improvements in neuromuscular coordination contribute to better swallowing outcomes by enhancing airway protection, bolus propulsion and pharyngeal clearance [21].

Speech production relies on a complex interplay of respiration, vocalisation, resonance and articulation, requiring precise coordination of multiple muscle groups. Different phonatory tasks engage distinct muscles, such as the OO for lip movements and the dorsum of the tongue and soft palate for velar sounds. While any training targeting one element of the head–neck–face system could theoretically yield benefits across the entire system, research suggests that targeted training paradigms with specific tasks may produce more effective results in dysphagia rehabilitation [22]. Studies indicate that motor training should focus on activating muscle groups involved in essential motor sequences rather than relying solely on generalised exercises. Although transfer effects from vocalisation exercises may enhance sensory‐motor function, improvements in non‐targeted muscle groups may be limited if the training lacks specificity [23]. However, in PSD patients, the precise muscle activation patterns associated with different phonatory tasks remain unclear, which has limited the development of more specific and sophisticated vocalisation‐based protocols for swallowing rehabilitation.

Thus, the present study aimed to analyse the correlation between muscle activity during phonatory tasks and during swallowing in PSD patients. Specifically, we used surface electromyography (sEMG) to compare the activation patterns of head muscles (OO and MS), submental muscles (SUB) and neck muscles (SCM) during vocalisation between PSD patients and healthy controls. The sEMG screening provides valuable insights into the oral, pharyngeal and initial oesophageal phases of swallowing, making it a reliable tool for assessing neuromuscular function [24, 25]. We also recorded swallowing‐related muscle activity during dry swallowing, which eliminates aspiration risk while effectively engaging all anatomical structures involved in the swallowing process [26]. We hypothesised that, in PSD patients, the mean sEMG amplitudes during phonatory tasks would correlate with the mean sEMG amplitudes during swallowing. We also expected the corrected mean sEMG values across different muscle groups during phonatory tasks to be distinct and significantly higher in PSD patients compared to healthy individuals. The results would foster structured intervention strategies for the precise rehabilitation of swallowing‐related muscle functions.

## 2 Materials and Methods

### 2.1 Participants

We enrolled 60 PSD patients at the First Affiliated Hospital of Soochow University between 1 August 2022, and 28 February 2024. Patients were eligible if they were at least 18 years old, had a prior stroke diagnosis and provided written informed consent. They also had a Mini‐Mental State Exam (MMSE) score of ≥ 21, a dysphagia diagnosis confirmed by a videofluoroscopic swallow study (VFSS) and no other conditions affecting their swallowing function. The control group consisted of 60 healthy volunteers who were at least 18 years old, had no history of dysphagia or neurological pathology and provided written informed consent. They had an MMSE score of ≥ 21, a Dysphagia Outcome and Severity Scale (DOSS) score of ≥ 7, no disorders affecting swallowing physiology, and maintained a normal diet. Subjects were excluded if they were unable to cooperate with surface electromyography (sEMG) testing, had a diagnosed speech disorder or dysarthria preventing phonatory tasks or exhibited unstable vital signs.

The appropriate sample size (*N*) for this study was determined using the following formula:

$$N={\left(\frac{Z_{\alpha }+Z_{\beta }}{C}\right)}^{2}+3$$

$$C=0.5\;\ln\;\frac{1+r}{1-r}$$

where *Z*_α_ and *Z*_β_ are the *Z* values corresponding to the significance level (α) and the probability of type II error (β), respectively, and *r* is the correlation coefficient. When α = 0.05 and β = 0.80, according to the Z‐score table, *Z*_α_ and *Z*_β_ take the values of 1.96 and 0.84, respectively. The correlation coefficient, referenced from relevant literature, is *r* = 0.459 [17]. Based on these parameters, the required sample size was *N* = 35. Therefore, the final number of participants in this study exceeded the calculated minimum, ensuring sufficient statistical power to analyse the influence of one variable on another.

### 2.2 **sEMG** Setup

A dual channel MyoTrac Infiniti System (Thought Technology Ltd., Montreal, Canada) was utilised for surface electromyography. The setup included disposable patch electrodes configured in pairs and a third electrode serving as the ground. Electrode placement for each muscle group was determined according to literature guidelines [27], with adjustments made as needed to accommodate anatomical variations [28]. Participants were seated upright in a chair with back support, and surface electrodes were securely applied to the skin in the head and neck regions. Before placement, the skin was thoroughly cleansed with a 70% isopropyl alcohol wipe. The specific locations of electrode placement are as follows:

- OO: At the right or left corner of the mouth.
- MS: Obliquely over the mid‐belly of MS, aligned with muscle fibres.
- SUB: On the skin beneath the chin, slightly to the right or left of the midline.
- SCM: Diagonally at about 50% of the distance between the mastoid process and sternal notch, parallel to SCM fibres.

The sEMG signals were recorded using BioGraph Infiniti 3.0 (Thought Technology). The signals underwent bandpass filtering (50–250 Hz), full‐wave rectification and were sampled at a frequency of 1000 Hz. System parameters included a common‐mode rejection ratio greater than 130 dB, a gain of 1000 and noise levels below 1 μV.

### 2.3 Tasks

#### 2.3.1 Resting

Participants were instructed to close their eyes, relax their jaw, slightly open their mouth and refrain from swallowing for 30 s with their eyes closed before formal measurements. Recording began once the sEMG signal maintained a stable baseline without significant fluctuations for more than 30 s.

#### 2.3.2 Phonation

The mean sEMG amplitudes were recorded while the participants performed the following phonatory tasks:

- **Pursed‐**lip breathing **(PLB)**: Participants voluntarily inhaled through the nose, allowing their abdominal muscles to relax and expand. During exhalation, they pursed their lips while engaging their abdominal muscles to exhale slowly and steadily [20].
- **End‐**inspiratory breath holding **(EIBH)**: Participants slowly inhaled through the nose, taking in minimal air. They held their breath for approximately 3 s before exhaling through the nose, extending the exhalation as much as possible while avoiding abdominal muscle contraction [29].
- **Lip** trills: Participants generated an audible vibrating sound by forcefully expelling air through closed but relaxed lips, creating a strong airflow [30].
- **Pronouncing /a/**: Participants voluntarily sustained the vowel sound /a/ for as long and as consistently as possible [31].
- Pronouncing /n/: Participants produced the nasal consonant /n/, with perceptible vibrations in the lips, jaw and nose during articulation [32].
- **Pronouncing /m/**: Participants produced the nasal consonant /m/ engaging the lips, jaw and nose, with vibrations perceptible during pronunciation [32].

#### 2.3.3 Swallowing

Participants performed voluntary single swallows of saliva, referred to as ‘dry’ swallows. Each participant completed three trials and was instructed to exert maximum effort in each attempt. The first trial served as a familiarisation exercise and was excluded from analysis, leaving the remaining two trials for assessment.

All tests were conducted and analysed by the same researcher to ensure consistency. The intraclass correlation coefficient (ICC) for the eight major parameters exceeded 0.9, indicating excellent intra‐rater reliability. For data analysis, the average of the two valid trials was used as the representative value.

### 2.4 **Data** Analysis

Statistical analyses were conducted using SPSS 23.0 and GraphPad Prism 9. The Shapiro–Wilk test was applied to assess the normality of quantitative data. Variables that followed a normal distribution were reported as mean ± standard deviation (x̄ ± SD), whereas non‐normally distributed data were presented as median with interquartile range (x̃, [P25, P75]). Categorical variables were described using count and percentage (n, %).

The general characteristics of the patient and control groups were compared using a two‐sample Student's t‐test for continuous variables and a Chi‐square test for categorical variables. The mean sEMG amplitude during the dry swallowing task was analysed using a two‐sample Student's t‐test or a Mann–Whitney U‐test. Statistical significance was defined as *p* < 0.05.

Spearman's correlation analysis was performed to examine the relationship between the mean sEMG amplitudes of dry swallowing and various phonatory tasks. Scatter plots were generated to visualise the correlations and the comparisons of mean sEMG amplitudes across the four muscle groups during different phonatory tasks. To account for multiple comparisons, the Holm–Sidak correction was applied alongside Mann–Whitney U tests. Additionally, multiple linear regression analysis was conducted to evaluate the impact of phonatory tasks on the activity of swallowing‐related muscle groups.

## 3 Results

### 3.1 sEMG Amplitude at Rest and During Dry Swallowing

The study included 60 patients with PSD (23 men and 37 women) and 60 healthy volunteers (26 men and 34 women). There were no statistically significant differences between the two groups regarding age, gender, height, weight, BMI (Table 1) or the resting sEMG amplitude of the four muscle groups (Table 2). However, the patients had significantly higher mean sEMG amplitude values than the healthy controls in performing dry swallowing (Table 2).

**TABLE 1:** Demographic information of study subjects.

Characteristics	Patient (*n* = 60)	Control (*n* = 60)	*p*
Age (years)	66.57 ± 9.80	62.27 ± 10.22	0.102
Sex (M/F)	23/37	26/34	0.577
Height (m)	1.67 ± 0.09	1.64 ± 0.09	0.286
Weight (kg)	62.03 ± 8.52	63.77 ± 8.45	0.432
BMI (kg/m^2^)	22.21 ± 2.26	23.41 ± 2.58	0.061

**TABLE 2:** Comparison of sEMG amplitude (μV) at rest and during dry swallowing.

Task/Muscle	Patient (*n* = 60)	Control (*n* = 60)	*p*
At rest			
MS^a^	6.75 ± 2.31	6.50 ± 1.19	0.592
OO^a^	6.91 ± 2.71	6.48 ± 1.95	0.487
SUB^a^	6.18 ± 2.09	5.58 ± 1.30	0.187
SCM^a^	4.87 ± 2.29	4.67 ± 1.60	0.698
Dry swallowing			
MS^b^	22.86 [16.38;29.16]	17.68 [16.17;23.34]	0.029*
OO^b^	19.05 [14.86;24.62]	16.64 [13.82;19.74]	0.039*
SUB^a^	21.08 ± 5.97	18.67 ± 4.81	0.036*
SCM^b^	9.95 [7.95;15.12]	7.98 [5.88;11.36]	0.022*

Abbreviations: MS, masseter; OO, orbicularis oris; SUB, submental muscle; SCM, sternocleidomastoid muscle.

^a^ Expressed as median [Q_1_, Q_3_].

^b^ Expressed as mean ± standard deviation.

* *p* < 0.05.

### 3.2 Comparison of sEMG During Tasks Between Patients and Controls

**FIGURE 1:**
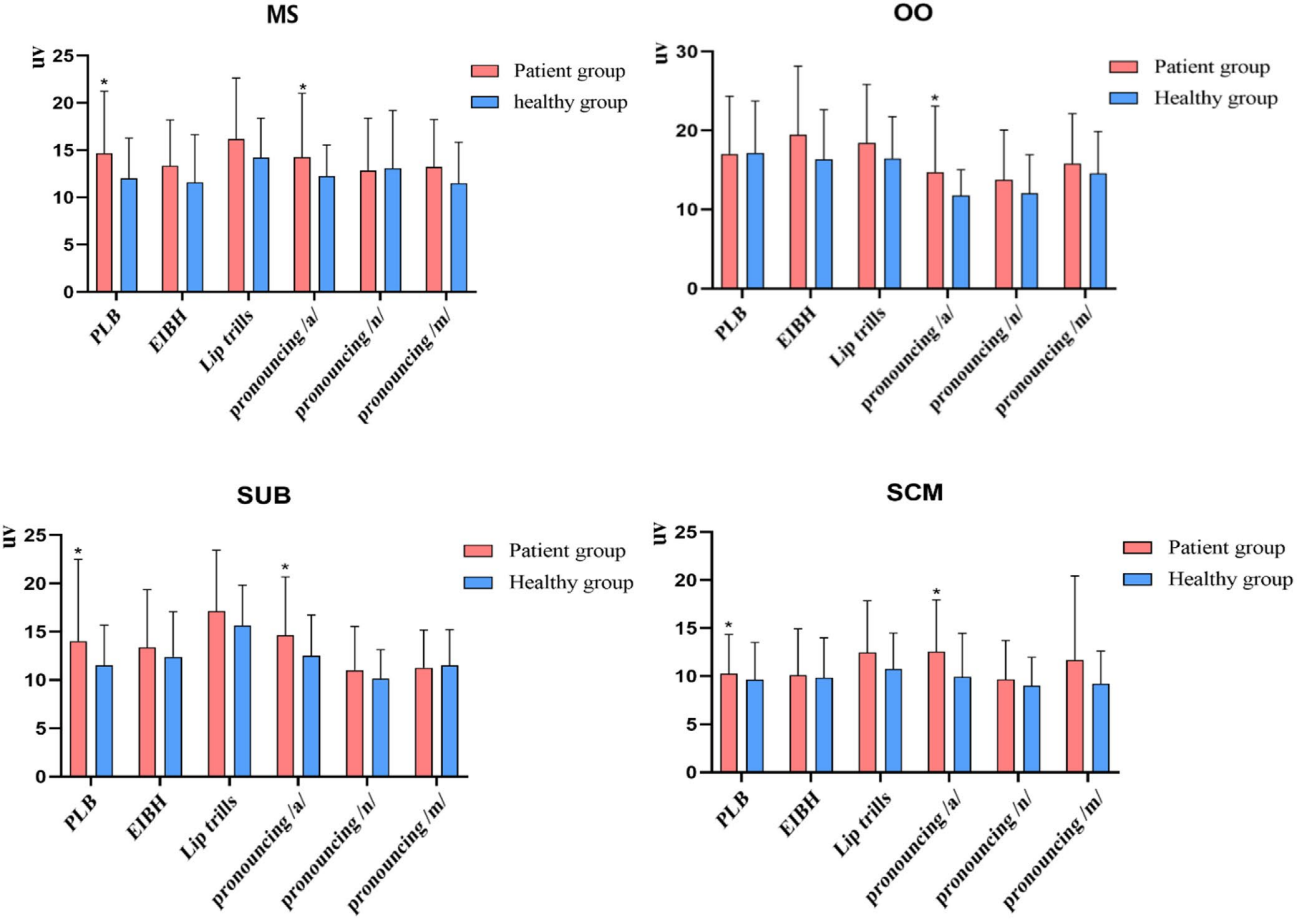
The mean sEMG amplitudes of the four muscle groups during different phonatory tasks.

The mean sEMG amplitudes during phonatory tasks were compared between the patients and the healthy controls (Figure 1). The patients had significantly elevated (*p* < 0.05) mean sEMG amplitudes in various muscles during different tasks, that is, MS, SUB and SCM during PLB, and MS, OO, SUB and SCM during the pronunciation of /a/.

### 3.3 **Correlations** Between the sEMG Signals During Dry Swallowing and During Phonatory Tasks

**FIGURE 2:**
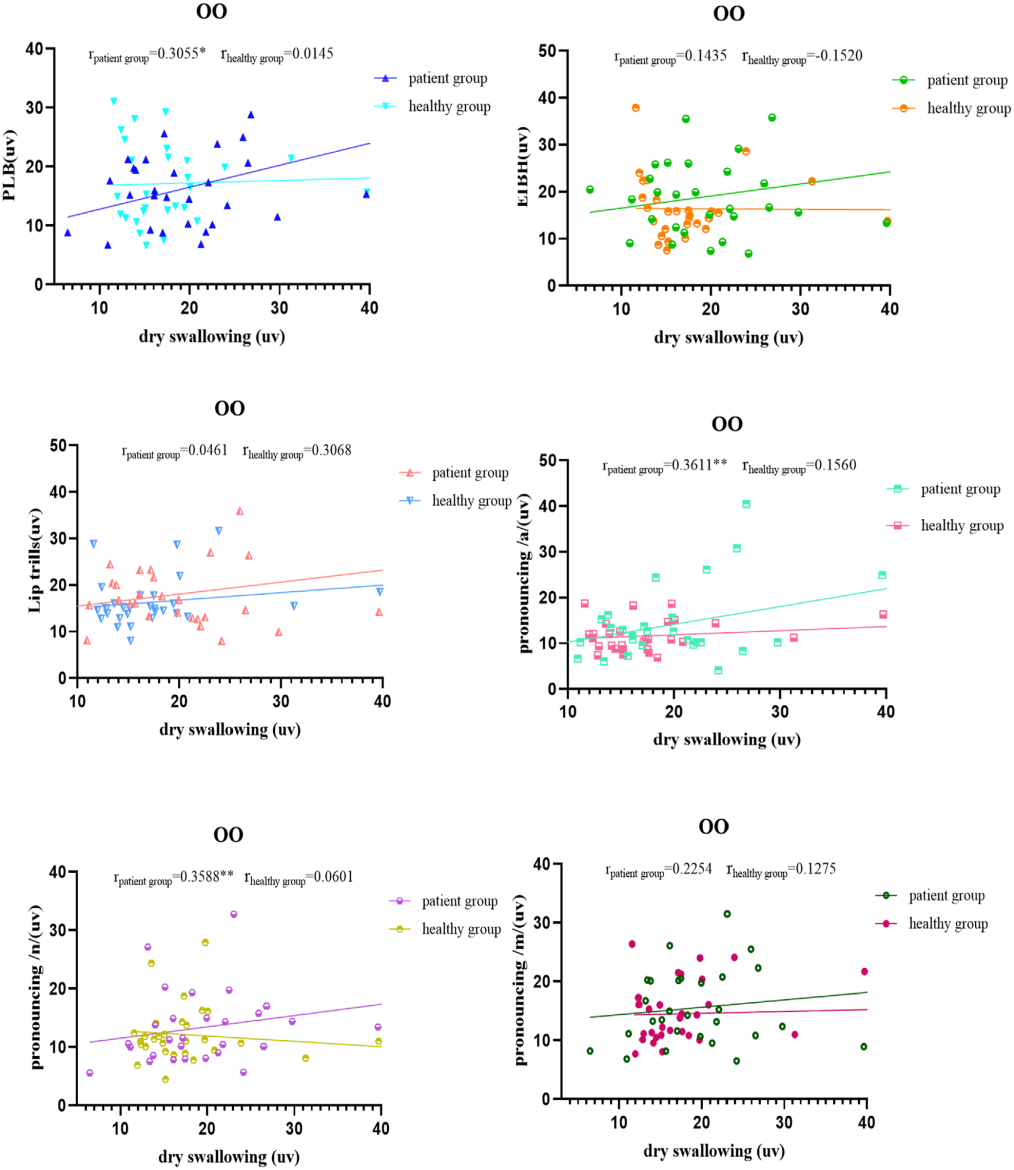
Correlation analysis of the sEMG amplitude of the orbicularis oris muscle (OO) during dry swallowing and during phonatory tasks.

**FIGURE 3:**
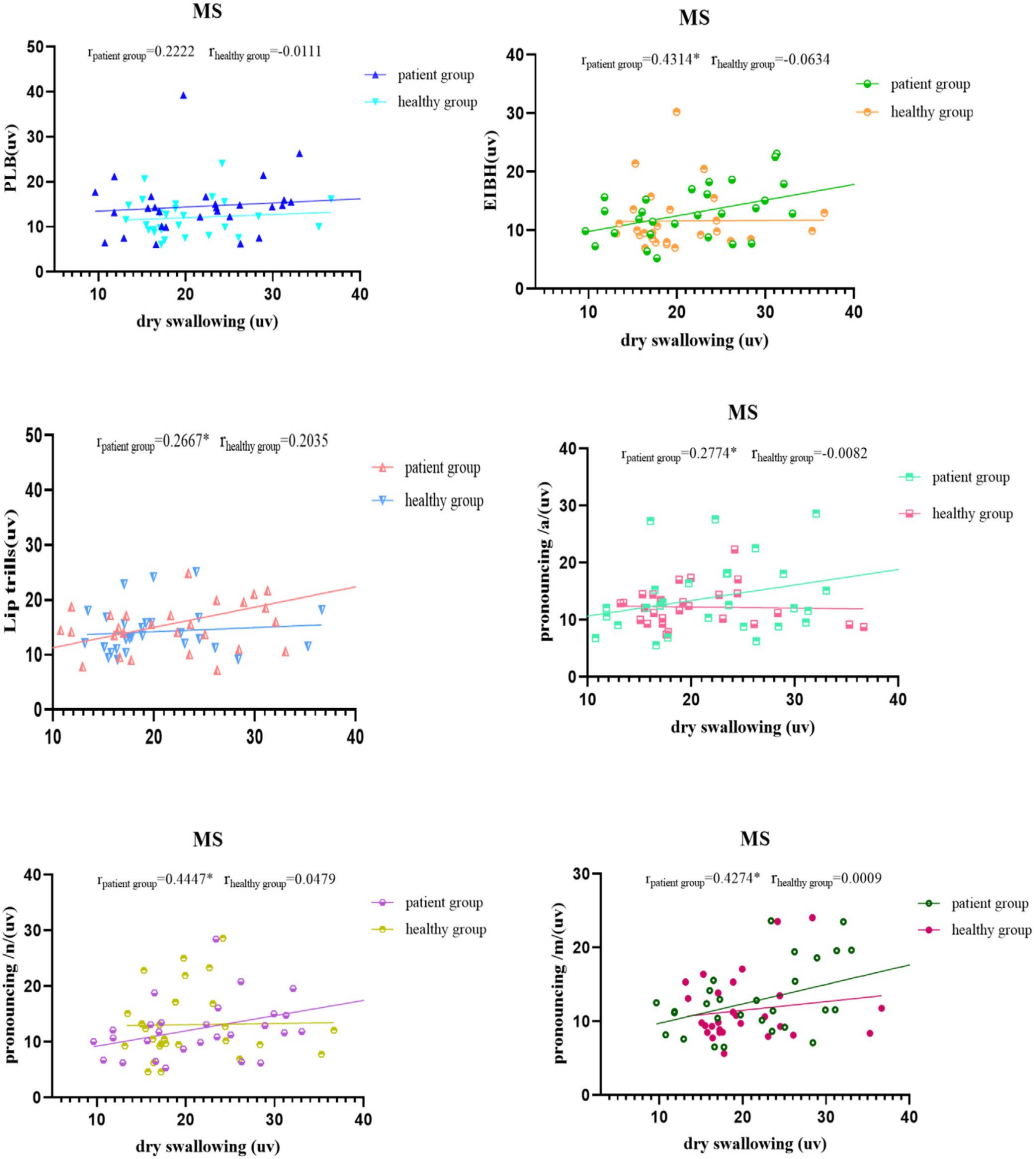
Correlation analysis of the sEMG amplitude of the masseter muscle (MS) during dry swallowing and during phonatory tasks.

**FIGURE 4:**
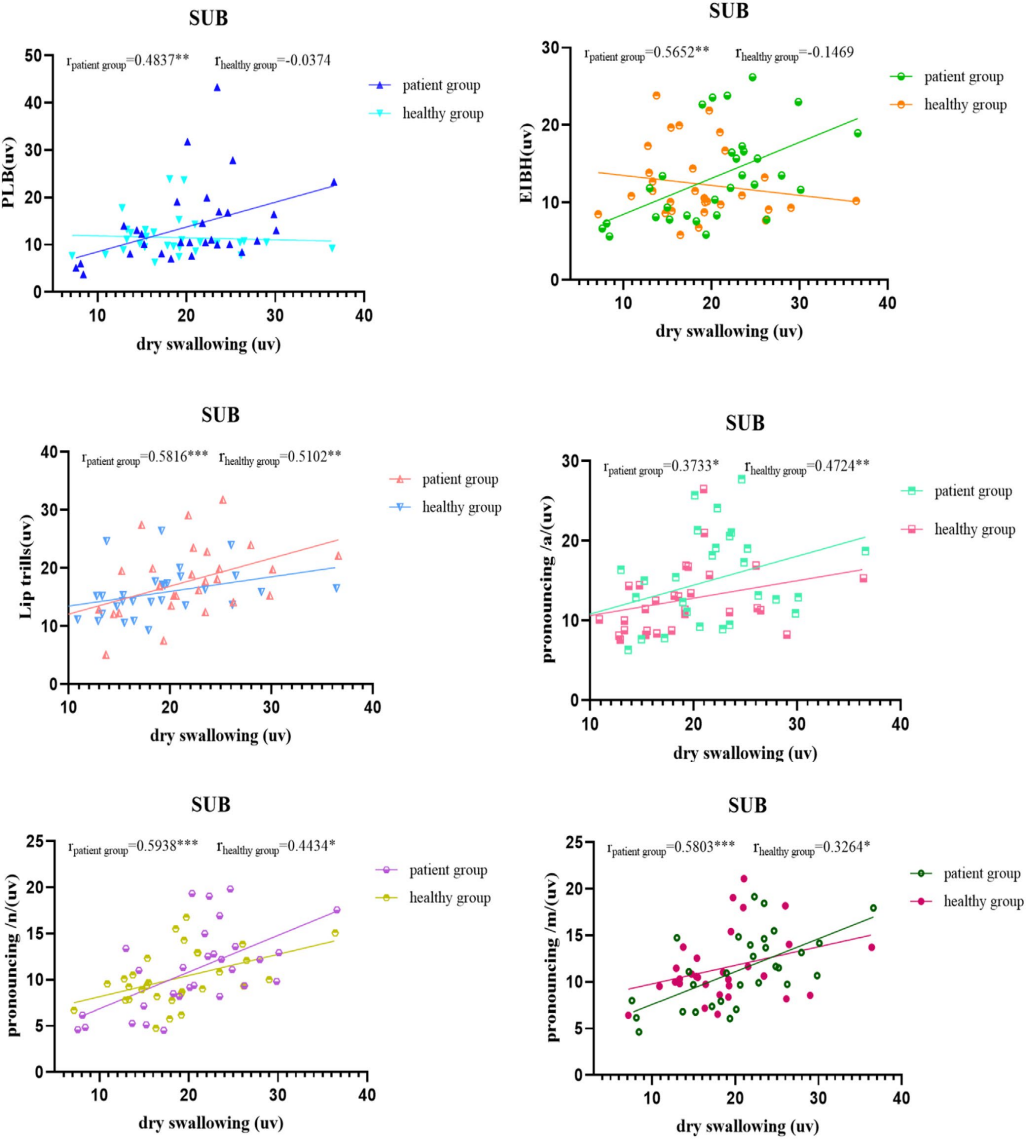
Correlation analysis of the sEMG amplitude of the submental muscle (SUB) during dry swallowing and during phonatory tasks.

**FIGURE 5:**
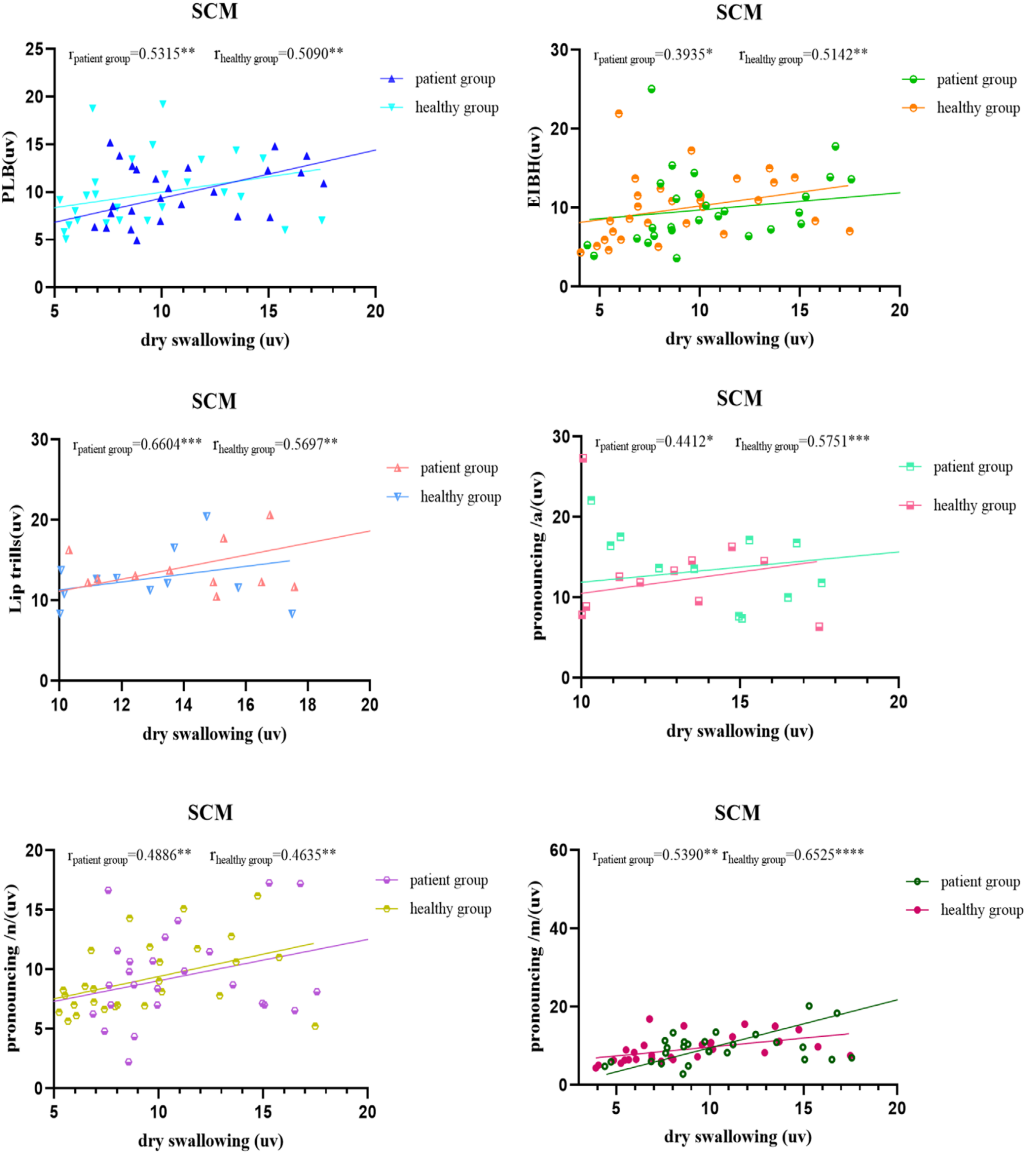
Correlation analysis of the sEMG amplitude of the sternocleidomastoid muscle (SCM) during dry swallowing and during phonatory tasks.

Figures 2, 3, 4, 5 and Table 3 present Spearman correlations between the mean sEMG amplitudes during phonatory tasks and during dry swallowing. The Spearman's rank correlation coefficient is a non‐parametric statistic that does not require any specific data distribution and is not sensitive to outliers. Therefore, it can effectively avoid the influence of outliers on the results.

**TABLE 3:** Correlations between the mean sEMG amplitudes of dry swallowing and phonatory tasks (expressed as r and 95% CI).

Muscle	Group	Phonatory task					
		PLB	EIBH	Lip trills	/a/	/n/	/m/
MS	Patient	0.2222 [−0.0412, 0.4568]	0.4314* [0.1919, 0.6224]	0.2667* [0.0061, 0.4935]	0.2774* [0.0176, 0.5021]	0.4447* [0.2077, 0.6324]	0.4274* [0.1872, 0.6193]
	Control	−0.0111 [−0.2714, 0.2507]	−0.0635 [−0.3193, 0.2010]	0.2035 [−0.0608, 0.4412]	−0.0082 [−0.2688, 0.2534]	0.0479 [−0.2159, 0.3052]	0.0009 [−0.2603, 0.2619]
OO	Patient	0.3055* [0.0482, 0.5247]	0.1435 [−0.1222, 0.3900]	0.0461 [−0.2177, 0.3035]	0.3611** [0.1104, 0.5686]	0.3588** [0.1079, 0.5668]	0.2254 [−0.0380, 0.4594]
	Control	0.0145 [−0.2476, 0.2745]	−0.1520 [−0.3973, 0.1136]	0.3068 [0.04971, 0.5258]	0.1560 [−0.1096, 0.4007]	−0.0601 [−0.3162, 0.2042]	0.1275 [−0.1382, 0.3761]
SUB	Patient	0.4837** [0.2548, 0.6613]	0.5652** [0.3568, 0.7200]	0.5816*** [0.3779, 0.7316]	0.3733* [0.1244, 0.5781]	0.5938*** [0.3937, 0.7401]	0.5803*** [0.3762, 0.7307]
	Control	−0.0374 [−0.2956, 0.2259]	−0.1469 [−0.3929, 0.1188]	0.5102** [0.2874, 0.6806]	0.4724** [0.2410, 0.6529]	0.4434* [0.2062, 0.6314]	0.3264* [0.0714, 0.5414]
SCM	Patient	0.5315** [0.3140, 0.6960]	0.3935* [0.1476, 0.5936]	0.6604*** [0.4825, 0.7859]	0.4412* [0.2035, 0.6297]	0.4886** [0.2608, 0.6649]	0.5390** [0.3235, 0.7014]
	Control	0.5090** [0.2859, 0.6798]	0.5142** [0.2930, 0.6839]	0.5697** [0.3626, 0.7232]	0.5751*** [0.3696, 0.7271]	0.4635** [0.2302, 0.6463]	0.6525**** [0.4718, 0.7806]

Abbreviations: /a/, /n/, /m/, pronunciation of the corresponding phoneme; EIBH, end‐inspiratory breath holding; MS, masseter; OO, orbicularis oris; PLB, pursed‐lip breathing; SCM, sternocleidomastoid muscle; SUB, submental muscle.

* *p* < 0.05.

** *p* < 0.01.

*** *p* < 0.001.

**** *p* < 0.0001.

In the healthy subjects, the sEMG amplitudes of MS and OO during dry swallowing did not show significant correlations with their amplitudes recorded during phonatory tasks. In the PSD patients, the sEMG amplitude of MS during dry swallowing was significantly correlated with its amplitudes recorded during all phonatory tasks except PLB, while the sEMG amplitude of OO during dry swallowing was significantly correlated with its amplitudes recorded during PLB, /a/ and /n/ tasks. Additionally, in the PSD patients, the sEMG amplitude of SUB during dry swallowing showed significant correlations with its amplitudes recorded across all tasks, whereas in healthy controls, the correlation was significant only during lip trill, /a/, /n/ and /m/ tasks. Across all subjects, the sEMG amplitude of SCM during dry swallowing was significantly correlated with its amplitudes recorded during all phonatory tasks.

### 3.4 Multiple Regression Analyses

Table 4 presents the results of multiple regression analyses for the patients. The muscle activity of OO during swallowing was significantly influenced by the PLB task (β = 0.686, *p* = 0.003). The muscle activities of MS (β = 0.300, *p* = 0.049), SUB (β = 0.297, *p* = 0.009) and SCM (β = 0.804, *p* = 0.001) during swallowing were significantly affected by lip trills. The muscle activities of OO (β = 0.299, *p* = 0.047) and SCM (β = 0.219, *p* = 0.047) during swallowing showed significant effects from pronouncing /a/. The muscle activities of SUB (β = 0.376, *p* = 0.041) and SCM (β = −0.503, *p* = 0.005) during swallowing were significantly influenced by pronouncing /n/. The muscle activity of SCM during swallowing was significantly affected by pronouncing /m/ (β = 0.281, *p* = 0.027).

**TABLE 4:** Multiple regression analyses of the mean sEMG amplitudes of dry swallowing and phonatory tasks in the patients.

Independent variables	MS			OO			SUB			SCM		
	*B*	*β*	*p*	*B*	*β*	*p*	*B*	*β*	*p*	*B*	*β*	*p*
PLB	−0.155	−0.103	0.403	1.004	0.686	0.003	0.045	0.056	0.678	0.071	0.054	0.789
EIBH	0.394	0.193	0.254	−0.328	−0.268	0.148	0.275	0.245	0.075	−0.071	−0.064	0.711
Lip trills	0.462	0.300	0.049^a^	−0.065	−0.045	0.834	0.317	0.297	0.009^a^	0.802	0.804	0.001^a^
/a/	0.156	0.106	0.474	0.382	0.299	0.047^a^	−0.325	−0.293	0.072	0.218	0.219	0.047
/n/	−0.02	−0.011	0.951	0.030	0.018	0.900	0.561	0.376	0.041	−0.666	−0.503	0.005^a^
/m/	0.381	0.193	0.277	−0.301	−0.179	0.283	0.314	0.182	0.328	0.372	0.281	0.027

Abbreviations: /a/, /n/, /m/, pronunciation of the corresponding phoneme; EIBH, end‐inspiratory breath holding; MS, masseter; OO, orbicularis oris; PLB, pursed‐lip breathing; SCM, sternocleidomastoid muscle; SUB, submental muscle.

^a^ Not significant for healthy controls.

Table 5 presents the results of multiple regression analyses for healthy controls. The muscle activity of OO during swallowing was significantly influenced by the PLB task (β = 0.367, *p* = 0.027). The muscle activity of SCM during swallowing was significantly affected by pronouncing /a/ (β = 0.309, *p* = 0.048). The muscle activity of SUB during swallowing showed a significant effect from pronouncing /n/ (β = 0.515, *p* = 0.011). The muscle activity of SCM during swallowing was significantly impacted by pronouncing /m/ (β = 0.602, *p* = 0.023).

**TABLE 5:** Multiple regression analyses of the mean sEMG amplitudes of dry swallowing and phonatory tasks in the healthy controls.

Independent variables	MS			OO			SUB			SCM		
	*B*	*β*	*p*	*B*	*β*	*p*	*B*	*β*	*p*	*B*	*β*	*p*
PLB	0.067	0.049	0.773	0.424	0.367	0.027	−0.141	−0.098	0.473	−0.353	−0.377	0.065
EIBH	−0.122	−0.106	0.529	−0.097	−0.080	0.741	−0.228	−0.178	0.210	−0.050	−0.057	0.732
Lip trills	0.305	0.220	0.292	0.410	0.303	0.137	0.346	0.242	0.094	0.287	0.294	0.178
/a/	−0.447	−0.253	0.203	0.664	0.291	0.077	0.262	0.184	0.248	0.247	0.309	0.048
/n/	−0.050	−0.053	0.799	−0.603	−0.344	0.091	1.032	0.515	0.011	−0.241	−0.197	0.455
/m/	0.307	0.231	0.202	−0.243	−0.170	0.308	−0.433	−0.268	0.268	0.645	0.602	0.023

Abbreviations: /a/, /n/, /m/, pronunciation of the corresponding phoneme; EIBH, end‐inspiratory breath holding; MS, masseter; OO, orbicularis oris; PLB, pursed‐lip breathing; SCM, sternocleidomastoid muscle; SUB, submental muscle.

## 4 Discussion

Our results agreed well with previous findings suggesting that damaged swallowing muscles demand higher activation to complete swallowing manoeuvres successfully [27]. We noted positive correlations between the mean sEMG amplitudes during phonatory tasks and those during swallowing. In addition, different phonatory tasks activated specific muscle groups to varying degrees, and PSD patients always required greater muscle activation than healthy individuals to perform the tasks. Presumably, training systems could enhance swallowing muscle function in PSD patients by targeting muscles and nerves in certain phonatory tasks.

For PSD patients, rehabilitative training through phonatory tasks appears beneficial, since articulatory movements could effectively stimulate impaired swallowing muscle groups. In phonatory tasks, respiration, phonation and resonance are the three subsystems that must operate in perfect coordination to effectively produce and shape speech sounds [33]. While language‐based and non‐language voice production both rely on phonatory processes, non‐language tasks such as humming or vowel prolongation do not require structured linguistic articulation, even though they still involve resonance and breath control [34]. Semi‐occluded vocal tract (SOVT) exercises, such as lip trills, involve laryngeal adjustments and have been shown to improve laryngeal and pharyngeal function, enhancing vocal efficiency and reducing strain on the vocal folds [35]. We also found that lip trills can help activate the swallowing muscles in the head and neck of PSD patients. Further research is warranted to explore the potential of SOVT exercises in enhancing swallowing function in dysphagia patients and maximising their therapeutic benefits.

While PLB is commonly used to enhance expiratory muscle function, EIBH serves as a modified form of inspiratory muscle training. Studies indicate that expiratory muscle training can significantly improve both lung capacity and swallowing function in patients; increased lung capacity contributes to better cough efficiency and enhanced airway protection [36, 37]. In contrast, while inspiratory muscle training primarily strengthens respiratory muscles and supports phonatory function, its direct benefits for swallowing function appear to be limited [37]. Our findings further demonstrated that PLB effectively stimulated the SUB muscle in PSD patients, whereas EIBH did not lead to significant activation of the swallowing muscle groups.

Language‐based phonatory tasks engage not only the larynx but also the coordinated activity of the lips, tongue and jaw, contributing to broader neuromuscular activation. In this study, articulation of the vowel /a/, a phoneme widely used in meaningful speech, was found to stimulate both laryngeal and head muscles in PSD patients. The LSVT protocol integrates intensive practice with /a/, along with other vocal exercises that emphasise loudness and sustained phonation [31]. While multiple components of LSVT contribute to improvements in maximal phonation time (MPT) and alternating movement rate (AMR) [19, 38], phonatory tasks specifically enhance vocal fold efficiency and resonance control [39], both of which are key elements linked to swallowing‐related muscle coordination [30, 40]. Enhanced MPT and AMR, whether stemming from phonatory exercises or broader vocal training, have been shown to correlate with improved swallowing function by strengthening orofacial and pharyngeal muscles, including SUB [15, 36, 37]. Moreover, research has demonstrated that SCM, which is typically classified as respiratory muscles within the perilaryngeal region, is also engaged in speech and responds dynamically to articulatory tasks [37, 41, 42, 43]. In this study, sEMG analysis revealed significantly higher SCM activation in PSD patients compared to healthy individuals during the articulation of /a/, suggesting a greater compensatory effort required for effective execution of this articulatory manoeuvre. This interplay between phonatory tasks and swallowing‐related muscle activation underscores the therapeutic potential of such tasks, reinforcing the need for continued investigation into their rehabilitative applications.

Resonance exercises, such as those involving the nasal consonant /n/, play a significant role in language training and pronunciation development. These exercises are primarily used for muscle relaxation, particularly in managing pitch‐related voice disorders and vocal nodules, as well as improving pronunciation quality, including enhancing vocal intensity in patients [32, 44, 45]. During training, resonance exercises are often combined with other phonatory tasks to maximise their effectiveness [45]. Depending on the additional pronunciation actions incorporated, resonance training can engage multiple muscle groups in the head and face, contributing to their activation and coordination.

In PSD patients, the sEMG signals of certain muscles recorded during specific tasks showed significant correlation with their signals recorded during dry swallowing, whereas no such correlation was observed in healthy controls (Table 3). Therefore, we conducted a multiple regression analysis on the swallowing‐related muscle groups of PSD patients and healthy controls. The results indicated that certain phonatory tasks had a significant impact on the swallowing‐related muscle groups of patients but no obvious effect on those of healthy controls (Tables 4 and 5). Lip trills affected the MS, SUB and SCM of the patients, but had no significant effect on those of healthy controls. Pronouncing /a/ affected the OO of the patients and pronouncing /n/ affected the SCM of the patients, but those tasks had no effect on the corresponding muscles of the healthy controls. This observation might be due to diminished motor control over head and neck muscles [46], as PSD patients must exert greater concentration, engage a broader range of muscle groups and apply more muscular strength than healthy individuals. Consequently, compensatory efforts are made, causing some phonatory tasks to affect the swallowing‐related muscle groups of the patients. It is possible that patients with oral‐phase dysphagia may benefit from targeted training in lip trills and the pronunciation of /a/, while those with pharyngeal‐phase dysphagia may benefit from lip trills, pronunciation of /a/ and resonance exercises. Tailored pronunciation‐based movement training, designed to strengthen impaired muscles, could provide a foundation for more precise and effective rehabilitation strategies for PSD patients.

Several limitations should be considered when interpreting the results of this study. First, validation in larger and more diverse cohorts is necessary to ensure generalisability to PSD patients with varying underlying conditions. Second, the variety of assessed tasks was limited, as complex phonatory tasks were excluded to avoid interference from fatigue, disease severity and aspiration risk. Lastly, this study captured only the immediate effects of swallowing muscle activity during phonation, and the dynamic changes over time were not determined. Future research should aim to expand participant diversity, incorporate a broader range of vocal manoeuvres and utilise longitudinal study designs to better track muscle activity progression during voice training.

## 5 Conclusions

This comparative study of sEMG in head and neck muscle movements among PSD patients revealed a significant correlation between the average sEMG amplitude during dry swallowing and that during various phonatory tasks. The sEMG profiles indicated that muscle activation varied across different muscle groups depending on the specific phonatory tasks performed. Thus, phonatory exercises could effectively enhance the recruitment of head and neck muscle strength in PSD patients, and specific phonatory movements may be translated into targeted clinical training protocols. These findings provide a potential clinical foundation for utilising precise phonatory movements to enhance the training of swallowing‐related muscle groups in PSD patients.

## Author Contributions

Conceptualisation, Y.Z., M.W.; methodology, Y.Z., M.W.; data collection, Y.Z., M.W.; data analysis, Y.Z., M.W.; writing – original draft, Y.Z., M.W.; writing – review and editing, J.C., C.N., W.Y., Y.D., Z.G.; funding acquisition, Y.Z., M.W.; supervision, Y.Z., M.W.; project administration, Y.Z., M.W.

## Ethics Statement

The study was approved by the Medical Ethics Research Committee of the First Affiliated Hospital of Soochow University. Informed consent was obtained from all participants.

## Conflicts of Interest

The authors declare no conflicts of interest.

## Data Availability

Additional data supporting the findings of this study are available from the corresponding authors upon reasonable request.
